# The effect of dexmedetomidine on gastric ischemia reperfusion injury in rats. Biochemical and histopathological evaluation

**DOI:** 10.1590/ACB360104

**Published:** 2021-02-01

**Authors:** Ufuk Kuyrukluyildiz, Leman Acun Delen, Didem Onk, Gulce Naz Yazici, Mine Gulaboglu, Halis Suleyman

**Affiliations:** 1 Associate Professor. Erzincan Binali Yıldırım University – Faculty of Medicine – Department of Anesthesiology and Reanimation – Erzincan, Turkey.; 2 PhD. Malatya Research and Training Hospital – Department of Anesthesiology and Reanimation – Malatya, Turkey.; 3 Associate Professor. Erzincan Binali Yıldırım University – Faculty of Medicine – Department of Anesthesiology and Reanimation – Erzincan, Turkey.; 4 Assistant Professor. Erzincan Binali Yıldırım University – Faculty of Medicine – Department of Histology and Embryology – Erzincan, Turkey.; 5 Professor. Ataturk University – Faculty of Pharmacy – Department of Biochemistry – Erzurum, Turkey.; 6 Professor. Erzincan Binali Yıldırım University – Faculty of Medicine – Department of Pharmacology – Erzincan, Turkey.

**Keywords:** Reperfusion Injury, Oxidative Stress, Dexmedetomidine, Stomach, Rats

## Abstract

**Purpose:**

To evaluate the protective effect of dexmedetomidine on gastric injury induced by ischemia reperfusion (I/R) in rats.

**Methods:**

A total of 18 male albino Wistar rats were divided groups as: gastric ischemia reperfusion (GIR), gastric ischemia reperfusion and 50 μg/kg dexmedetomidine (DGIR) and sham operation (HG) group. After the third hour of reperfusion, the biochemical and histopathological examinations were performed on the removed stomach tissue.

**Results:**

Malondialdehyde (MDA) and myeloperoxidase (MPO) levels were found to be significantly higher in GIR compared to HG (p < 0.05). A statistically significant decrease was observed at the DGIR compared to the GIR for oxidants levels. Total glutathione (tGSH) and superoxide dismutase (SOD) levels were statistically significantly decreased at the GIR, and antioxidants levels were found to be significantly higher in the DGIR (p < 0.05) There was no significant difference between HG and DGIR in terms of SOD (p = 0.097). The DGIRs’ epitheliums, glands and vascular structures were close to normal histological formation.

**Conclusions:**

Dexmedetomidine is found to prevent oxidative damage on the stomach by increasing the antioxidant effect. These results indicate that dexmedetomidine may be useful in the treatment of ischemia-reperfusion-related gastric damage.

## Introduction

The stomach damage caused by ischemia reperfusion (I/R) is an important clinical problem associated with various physiopathological events^1^. It is known that many hemorrhagic conditions including peptic ulcer bleeding, hemorrhagic shock, vascular rupture, and surgery may lead to gastric I/R^2^. The ischemic damage is defined as the pathological changes that occur due to the deprivation of oxygen in tissues or organs as a result of a decrease or complete interruption of the blood flow to the tissues, for various reasons^3^. Reperfusion is the restoration of the blood flow to the ischemic tissues^4^. If the blood flow to the ischemic tissue is not restored, a series of pathological events, which may progress to cellular dysfunction and cell necrosis, develop^5^. However, paradoxically,it has been reported that rapid reperfusion of ischemic tissue can cause much more severe damage compared to the damage caused by ischemia alone^4^. The reperfusion injury is caused by overproduction of reactive oxygen radicals (ROS), known as reperfusion mediators, by molecular oxygen, which are presented to ischemic tissue in large quantities by arterial blood^6^,^7^. These ROS produce toxic products such as malondialdehyde (MDA) from lipids byoxidizing cell membrane lipids. Another mechanism that causes tissue damage is the activation of cyclogenase-2 enzyme (COX-2) due to the increased intracellular calcium during ischemia and the release of proinflammatory prostaglandins and ROSs from arachidonic acid^4^.

This information summaries the pathogenesis of gastric I/R injury and the importance of antioxidant and anti-inflammatory drugs for treatment.This study examined the protective effectiveness of dexmedetomidine, which is an alpha-2 adrenergic receptor agonist, in I/R damage of the stomach. Dexmedetomidine inhibits sympathetic activity by presynaptic activation of alpha-2 adrenoreceptors in the central nerve system and causes decreased blood pressure and heart rate, sedation, and anxiolysis. In addition, it provides analgesia via alpha-2 adrenoreceptors in the spinal cord^8^.

Dexmedetomidine is also known to protect stomach tissue from indomethacin damage, due to its antioxidant activity^9^. It has been reported that dexmedetomidine inhibits the induction of COX-2 and other proinflammatory cytokines^10^. These information indicate that dexmedetomidine may protect the stomach from oxidative and inflammatory damage associated withI/R. The aim of this study is to investigate the effect of dexmedetomidine on I/Rinduced gastric injury in rats, biochemically and histopathologically.

## Methods

A total of 18 male albino Wistar rats weighing 260-280 g were used in the experiment. They were obtained from Atatürk University Medical Experimental Practice and Research Center. The animals were housed in groups at normal room temperature (22°C) under suitable conditions and fed before the experiment. Animal experiments were performed in accordance with the National Guidelines for the Use and Care of Laboratory Animals and were approved by the local animal ethics committee of Atatürk University, Erzurum, Turkey (Ethics Committee Number: 16 Dated: 26.12.2019).

### Chemicals

Sodium thiopental (IE-Ulagay,Istanbul) and dexmedetomidine (Abbott Co., UK.) were used for this evalution.

### Experimental groups

Before the experiment, 18 animals were divided into three equal groups.Each group included six male albino Wistar rats. The groups were named asgastric ischemia reperfusion group (GIR), gastric ischemia reperfusion group (DGIR)that was induced and 50 μg/kg dexmedetomidine was administered, and healthy group(HG).

### Experiment procedure

In order to carry out this experiment, 50μg/kg dexmedetomidine was administered intraperitoneally (i.p.) to the DGIR animal group. Distilled water was administered as solvent to the GIR and HG groups with the same volume and method. Thirty minutes after the administration of dexmedetomidine and distilled water, 25 mg/kg of thiopental sodium was injected into all rat groups (i.p.) and anesthesia was achieved by making therats breath xylazine at appropriate intervals. Afterthe thiopental sodium injection, the rats were kept waiting for an appropriate period for surgery. The appropriate period for surgical intervention is considered when the animals remain immobile in the supine position^11^. Then, a laparotomy with a 2.5 cm midline incision was applied to the rats, under sterile conditions. In order to induce ischemia reperfusion lesions, the celiac artery was clamped with clips to create ischemia for 1 hin the DGIR and GIR groups. The abdominal region of the HG group was opened without applying clips to the celiac artery and was closed by suturing. After 1 h, the clip was removed and reperfusion was achieved for 3 h^12^. At the end of the third hour of reperfusion, all animals were sacrificed with high dose (50 mg/kg) thiopenteal anesthesia. Then, biochemical and histopathological examinations were performed on the stomach tissue removed from the animals.

### The biochemical analysis

#### Determination of MDA

Determination of MDA is based on measuring the absorbance of the pink colored complex formed by thiobarbituric acid (TBA) and MDA at high temperature (95 °C), spectrophotometrically at a wavelength of 532 nm^13^. Homogenates were centrifuged at 5000g for 20 min, and these supernatants were used to quantify MDA. Then, 250 μL homogenate, 100 μL 8% sodium dodecyl sulfate (SDS), 750 μL 20% acetic acid, 750 μL 0.08% TBA, and 150 μL distilled water were pipetted into capped test tubes and vortexed. After the mixture was incubated at 100 °C for 60 min, 2.5 mL of n-butanol was added on it and spectrophotometrically measured. The amount of red color formed was determined by using 3-mL cuvettes at 532 nm, and the MDA amount of the samples was determined by using the standard graphic created using the MDA stock solution prepared before, by considering the dilution coefficients.

#### The determination of myeloperoksidaze (MPO) activity

The MPO activity was measured according to the modified method of Bradley *et al*.^14^. The homogenized samples were frozen and centrifuged at 1500 g for 10 min at 4 °C. The MPO activity in the supernatants were determined by adding 100 mL of the supernatant to 1.9 mL of 10 mmol/L phosphate buffer (pH equal to 6.0) and 1 mL of 1.5 mmol/L o-dianisidine hydrochloride containing 0.0005% (wt/vol) hydrogen peroxide. The changes in absorbance at 450 nm of each sample were recorded on anultraviolet-visible (UV-Vis)spectrophotometer.

#### The determination of tGSH

5,5’-dithiobis (2-nitrobenzoic acid) (DTNB) in the measurement medium is a disulfide chromogen and is easily reduced by sulfhydryl group compounds. The resulting yellow color was spectrophotometrically measured at 412 nm^15^. Homogenates were centrifuged at 12000 gfor 10 minand supernatants were used to determine the amount of GSH. Then, 1500 μL of measurement buffer (200 mmol/L Tris-HCl containing 0.2 mmol/L EDTA, pH = 8.2), 500 μL of supernatant, 100 μL of DTNB, and 7900 μL of methanol were vortexed by pipetting in capped test tubes. The mixture was left to incubate for 30 min at 37 °C and, then, measurements were made by spectrophotometer. The amount of yellow color formed was read by using 3-mL quartz cuvettes at 412 nm, and the GSH amount of the samples was determined by using the standard graphic created using the GSH stock solution prepared before, by considering the dilution coefficients.

### The superoxide dismutase (SOD) analysis

Measurements were performed according to the method of Sun *et al*.^16^. When xanthine is converted into uric acid by xanthine oxidase, SOD forms. If nitroblue tetrazolium (NBT) is added to this reaction, SOD reacts with NBT and a purple-colored formazan dye occurs. The sample was weighed and homogenized in 2 mL of 20 mmol/L phosphate buffer containing 10 mmol/L EDTA at pH 7.8. The sample was centrifuged at 6000 rpm for 10 min and, then, the brilliant supernatant was used as assay sample. The measurement mixture containing 2450 μL measurement mixture (0.3 mmol/L xanthine, 0.6 mmol/L EDTA, 150μmol/L NBT, 0.4 mol/L Na_2_CO_3_, 1 g/L bovine serum albumin), 500 μL supernatant and 50 μL xanthine oxidase (167 Unit/Liter) was vortexed. Then it was incubated for 10 min. At the end of the reaction, formazan was formed. The absorbance ofthe purple-colored formazan was measured at 560 nm. As the quantity of the enzyme increases,the quantity of oxygen radicals reacting with NMT decreases.

### The histopathological examination

All tissue samples were first identified in a 10% formaldehyde solution for light microscope assessment. Following the identification process, tissue samples were washed under tap water in cassettes for 24 h. Samples were then treated with conventional grade of alcohol (70, 80, 90 and 100%) to remove the water within tissues. Tissues were then passed through xylol and embedded in paraffin. Four-to-five micron sections were cut from the paraffin blocks and hematoxylin-eosin staining was administered. Their pictures were taken following the Olympus DP2-SAL firmware program (Olympus Inc. Tokyo, Japan) assessment. For semiquantitative analysis of histopathological examinations were evaluated as mucosal degeneration, dilatation, congestion, polymorphonuclear cell infiltration, mucosal edema and scored between 0 to 3. The histopathological assessment was carried out by the histologist blind for the study groups.

### The statistical analysis

Statistical analyses were conducted by using IBM SPSS Statistics for Windows, version 19 (Armonk, NY: IBM Corp.). Descriptive statistics were calculated for each variable.The results were presented as mean ± standard deviation (SD) for continuous variables. The significance of the variations between the groups was determined by using the one-way variance analysis (ANOVA) method, followed by the analysis by Tukey’s test. A value of p < 0.05 was considered statistically significant. While comparing groups for histopathological grades Kruskal-Wallis test was used and Dunn’s test was applied as post hoc.

## Results

### The biochemical results

The values of all study groups are shown comparatively in Table 1. The detailed analysis of each parameters is explained below.

**Table 1:** Biochemical results of the study groups.

		HG (n=6)				GIR (n=6)				DGIR (n=6)		
		Mean ± SD		Median (Min–Max)		Mean ± SD		Median (Min–Max)		Mean ± SD		Median (Min–Max)
MDA (μmol/g protein)		5.19 ± 0.29		5.15 (4.13-5.99)		9.94 ± 0.06*		9.96 (9.70-10.18)		5.73 ± 0.17**		5.87 (4.88-5.99)
MPO (U/g protein)		4.32 ± 0.07		4.28 (4.12-4.65)		9.13 ± 0.06*		9.13 (8.86-9.34)		5.10 ± 0.09**,+		5.16 (4.68-5.32)
tGSH (nmol/g protein)		7.65 ± 0.07		7.69 (7.41-7.84)		3.25 ± 0.11*		3.14 (3.11-3.85)		6.61 ± 0.08**,+		6.71 (6.26-6.82)
SOD (U/g protein)		19.50 ± 0.76		19.50 (17.0-22.0)		8.26 ± 0.27*		8.60 (7.10-8.80)		17.21 ± 0.95**		18.20 (13.7-19.2)

* p<0.001 compared to HG.

** p<0.001 compared to GIR.

+ p<0.001 compared to HG.

HG: Healthy group GIR: Gastric ischemia reperfusion group; DGIR: Gastric ischemia reperfusion + dexmedetomidine 50 μg/kg group.

Malondialdehyde levels were found to be significantly higher in the GIR ischemia reperfusion group (9.94 ± 0.06 μmol/g) compared to the HG group (5.16 ± 0.29 μmol/g). A statistically significant decrease was observed in the DGIR group compared to the GIR group (p < 0.05). There was no significant difference between the DGIR and HG groups in terms of MDA levels.

**Figure 1:**
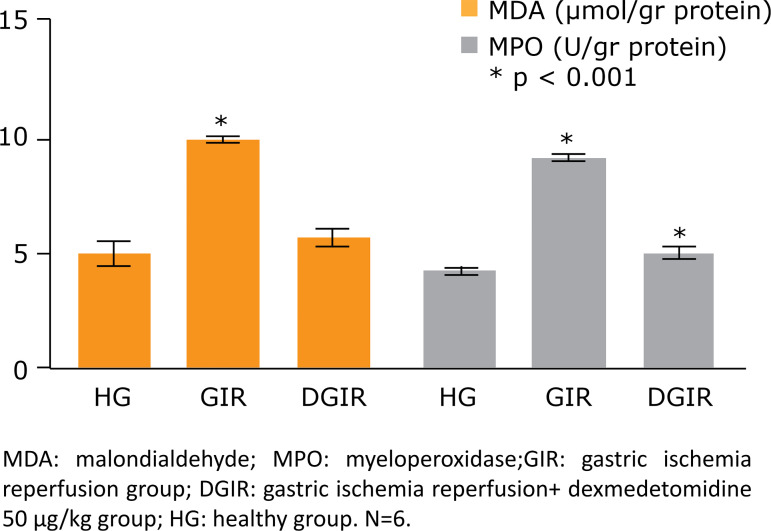
The amounts of MDA and MPO levels on stomach tissue at experimental groups. Bars are mean ± SD. The healthy group is compared with GIR and DGIR groups.

Myeloperoxidaselevels were found to be significantly higher in the GIR group (9.13 ± 0.06 μmol/g) compared to the HG group (4.32 ± 0.07 μmol/g). The decrease in MPO levels was statistically significant in the DGIR group compared to the GIR group (p < 0.05) (Fig.1).

Total glutathione levels were found to be 7.65 ± 0.07nmol/g in the HG group, while it was found to be 3.25 ± 0.11nmol/g in the GIR group; this difference was statistically significant (p < 0.05). In the DGIR group, tGSH levels were found to be 6.61±0.08 nmol/g and it was statistically significant compared to both GIR and HG groups (p < 0.05).

**Figure 2:**
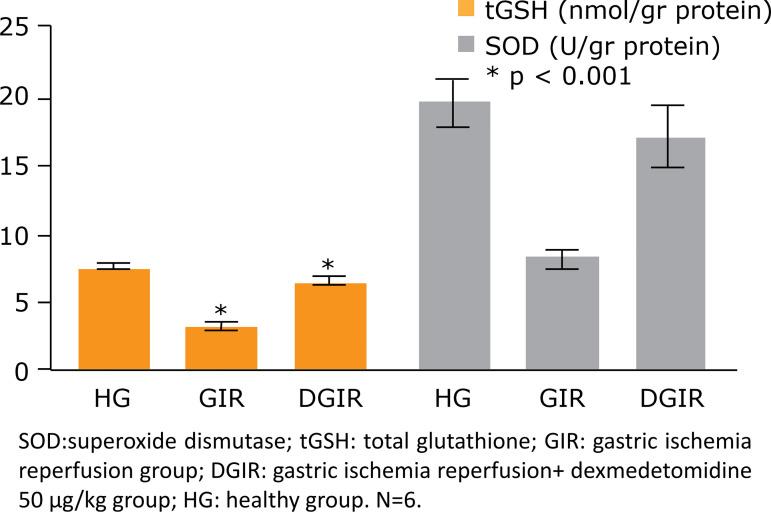
The amounts of tGSH and SOD levels on stomach tissue at experimental groups. Bars are mean ± SD. The healthy group is compared with GIR and DGIR groups.

When the SOD levels were compared, a statistically significant decrease was observed in the GIR group (8.26 ± 0.27 U/g) compared to the HG group (19.5 ± 0.76 U/g) (p < 0.05). Superoxide dismutase levels were found to be significantly higher in the DGIR group (17.2 ± 0.95 U/g) compared to the GIR group (p < 0.05). There was no significant difference between the HG and DGIR groups in terms of SOD levels (p = 0.097) (Fig. 2).

### The histopathological findings

**Figure 3:**
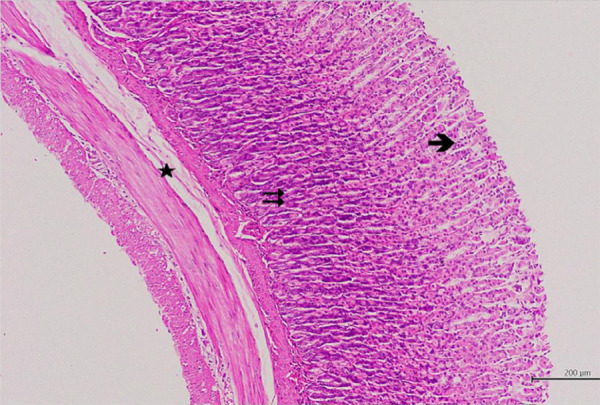
Hematoxylin–eosin staining in gastric tissue inhealthy control group; →: epithelium, ⇉: gastric glands, ★: blood vessels, 100×.

**Figure 4:**
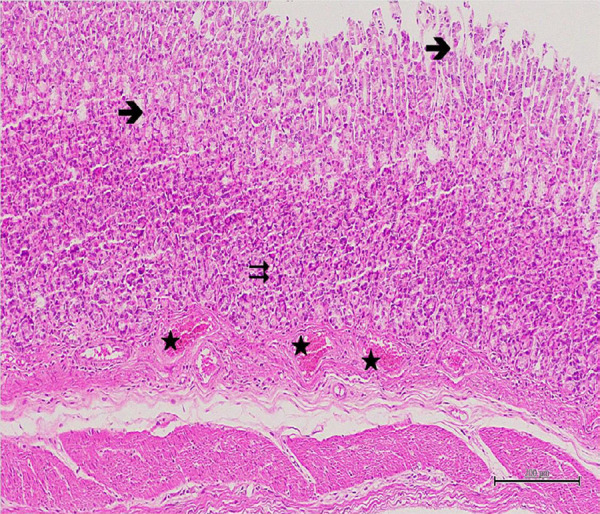
Hematoxylin–eosin staining in gastric tissuein GIR group; →: degenerated and shed epithelium, ⇉: edematous gastric glands, ★: congested and dilatated blood vessels, 100×.

**Figure 5:**
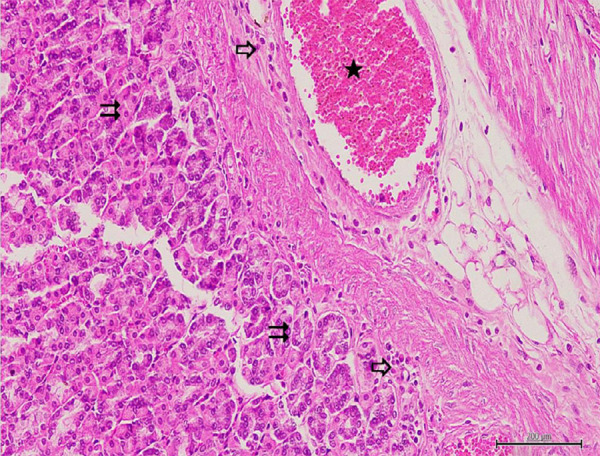
Hematoxylin–eosin staining in gastrictissue in GIR group; ⇉: edematous gastric glands, ⇨: polymorphonuclear cell infiltration,★: congested and dilatated blood vessels, 200×.

**Figure 6:**
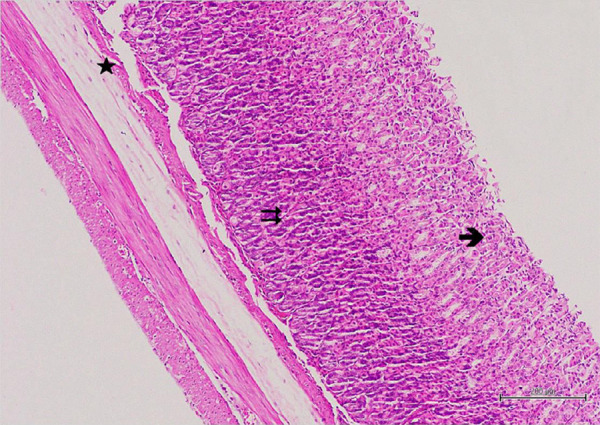
Hematoxylin–eosin staining in gastric tissue in DGIR group; →: epithelium, ⇉: gastric glands, ★: mild dilatated blood vessels, 100×.

The scores of histopathological assessments are shown at Table 2.The healthy control (HG) group showed normal histological architecture of the gastric tissue, surface epithelium, glands of the tunica mucosa, and the gastric stratification (Fig. 3). In the examination of the GIR group, it was observed that the surface epithelium was torn off in places, the gland recesses were reduced, the neck areas of the glands were opened, the base regions were edematous, and the blood capillaries were intenselydilatated and congested (Fig. 4). In addition, in the samples belonging to this group, polymorphonuclear cell infiltration was noted in the connective tissue area adjacent to the vascular and gland bases (Fig. 5). When the samples belonging to the DGIR group were evaluated, it was found that epithelium, gland and vascular structures were close to normal histological formation, regeneration developed in the epithelium, edema regressed in the gastric glands,The polymorphonuclear leukoycte (PMNL) cells substantially reduced. Blood vessels were generally normal also some blood vessels were mildly dilatated (Fig. 6).

**Table 2:** Histopathological examination of the gastric tissues in study groups.

Mucosal degeneration		Groups						p
		HG		GIR		DGIR		
		0.0 ± 0.0		2.56 ± 0.31a		0.50 ± 0.11		< 0.001
Dilatation		0.0 ± 0.0		2.58 ± 0.29a		0.22 ± 0.25b		0.001
Congestion		0.0 ± 0.0		2.72 ± 0.14a		0.33 ± 0.10		< 0.001
Polymorphonuclear cell infiltration		0.0 ± 0.0		2.53 ± 0.29a		0.19 ± 0.19b		0.001
Mucosal edema		0.0 ± 0.0		2.64 ± 0.16a		1.11 ± 0.52		< 0.001

Results were presented as mean±SD. Kruskal–Wallis test was performed when comparing groups. For pairwise comparisons Dunn’s test was used. aStatistically significant (p < 0.05) when compared with HG, bwhen compared with GIR.

## Discussion

Alpha-2adrenergic receptor (α2-AR) agonists have been used successfully in many clinical settings, considering various actions including sedation, analgesia, anxiolytic, perioperative sympatholytic, cardiovascular stabilizing effects, reduced anesthetic requirements, and preservation of respiratory function^17^. Anti-inflammatory and antioxidant activities have also been reported in the literature. Thanks to its wide spectrum of action and side effects limited to hemodynamic effects, dexmedetomidine have been used for many clinical applications as premedication,intraoperative use,locoregional anesthesia, procedural sedation,controlled hypotension etc^17^. There are many studies investigating the effectiveness of dexmedetomidine especially on the repairment of kidney, lung, liver and brain tissue damages^18^-^22^. However, in the literature, there are a limited number of studies on the protective effects of dexmedetomidine on stomach.

In the study of Chen *et al*.^18^, it was found that dexmedetomidine improved renal dysfunction, reduced oxidative stress, suppressed apoptosis, and decreased ROS formation by inhibiting noradrenaline release. Therefore, they experimentally demonstrated that dexmedetomidine was protective against stress-inducing acute kidney damage by suppressing apoptosis and reducing oxidative stress. In the study of Güzel *et al.* 19, anti-inflammatory and antioxidant effect of dexmedetomidine has been shown to suppress the harmful effects of HCL-related acute lung injury experimentally induced in the lung. Sha *et al*.^20^ demonstrated the effectiveness of dexmedetomidine in reversing liver functions by its antioxidant and antiapoptotic effects on oxidative stress.It was also shown to reduce inflammation and apoptosis in heart tissue via AMPK-GSK3 BETA and was recommended in surgical patients with heart diseases^21^. Huang *et al*.^22^ found that propofol and dexmedetomidine have different antineuroinflammatory and neuroprotective effects.

In a study conducted in an *in vitro* environment in 2016, it was observed that dexmedetomidine increased SOD levels and decreased MDA levels^23^.In this study, it was also observed that dexmedetomidine applied after experimentally induced ischemia-reperfusion in the stomach has an antioxidant effect. In addition, oxidant parameters MDA and MPO were found to be low,while the antioxidant parameters tGSH and SOD levels were high,in the dexmedetomidine group. Dexmedetomidinehas been reported to display its anti-inflammatory effect by inhibiting the induction of COX-2 and other proinflammatory cytokines.

Dexmedetomidine is also known to protect stomach tissue from indomethacin damage due to its antioxidant activity. Polat *et al*.^9^showed that dexmedetomidine increases antioxidant parameters and decreases oxidant enzymes, and suggested that the antiulcerative action mechanism of dexmedetomidine is due to this antioxidant activity. In this study, overproduction of reactive oxygen radicals induced by the reperfusion of the ischemic tissue was found to be lower in the DGIR group compared to the GIR group, and histopathological examination of the tissueswas similar to the HG group.

Ina study investigating the anti-inflammatory activity of dexmedetomidine on the liver and intestine, dark eosinophilic cytoplasm andhepatocytes with heterochromatic nuclei in liver sections were rare and a limited inflammation in the local area was present, in the dexmedetomidinegroup; and a significant decrease in histopathological damage scoring was observed^24^. It was reported that dexmedetomidine reduced oxidative stress in organs and corrected histopathological changes in liver. In this study, it was found that the epithelial, glandular, and vascular structures in the DGIR group after ischemia reperfusion were similar to the control group^25^. The dexmedetomidine corrected the damage caused byexperimental ischemia-reperfusion in the stomach by regulating the oxidant-antioxidant balance was found at our study. This biochemical improvement was also confirmed by histopathological examination of the stomach tissue.

Current evidence suggests that disruption of the oxidative mechanism of cells and changes of ATP levels in tissue may be the basis of tissue damage^25^. The results of our research findings supported this view especially increased oxidative stress caused damage to the stomach tissue. Administration of dexmedetomidine turned the oxidative balance in favor of antioxidants and prevented tissue damage.

## Conclusions

It has been biochemically and histopathologically shown that ischemia-reperfusion process causes oxidative damage in gastric tissue. On the other hand, dexmedetomidine was found to prevent oxidative damage in the stomach by increasing the antioxidant effect. These results indicate that dexmedetomidine may be useful in the treatment of ischemia-reperfusion-related gastric damage.
